# The Paradigm Shift From Patient to Health Consumer: 20 Years of Value Assessment in Health

**DOI:** 10.2196/60443

**Published:** 2025-01-10

**Authors:** Eline M van den Broek-Altenburg, Adam J Atherly

**Affiliations:** 1 University of Vermont Burlington, VT United States; 2 Virginia Commonwealth University Richmond, VA United States

**Keywords:** value assessment, cost-effectiveness, quality-adjusted life-years, QALY, health consumer, health technology, value based, digital health, patient centered, preferences, health economics

## Abstract

Health care is undergoing a “revolution,” where patients are becoming consumers and armed with apps, consumer review scores, and, in some countries, high out-of-pocket costs. Although economic analyses and health technology assessment (HTA) have come a long way in their evaluation of the clinical, economic, ethical, legal, and societal perspectives that may be impacted by new technologies and procedures, these approaches do not reflect underlying patient preferences that may be important in the assessment of “value” in the current value-based health care transition. The major challenges that come with the transformation to a value-based health care system lead to questions such as “How are economic analyses, often the basis for policy and reimbursement decisions, going to switch from a societal to an individual perspective?” and “How do we then assess (economic) value, considering individual preference heterogeneity, as well as varying heuristics and decision rules?” These challenges, related to including the individual perspective in cost-effectiveness analysis (CEA), have been widely debated. Cost-effectiveness measures treatments in terms of costs and quality-adjusted life-years (QALYs), where QALYs assume that a health state that is more desirable is more valuable, and therefore, value is equated with preference or desirability. QALYs have long been criticized for empirical and conceptual shortcomings. However, policy makers in many countries have used QALY measures to make health coverage decisions, although now, patients, and patient advocates, are questioning the valuation methodologies. This has led to the development of new approaches to valuing health, which are already starting to be used in the United States. This paper reviews 20-25 years of value assessment approaches in health and concludes with challenges and opportunities for value assessment methods in health in the years to come.

## Background

The concept of value assessment has been used for decades in marketing, management, and other fields to develop strategies to assess the potential and realized customer value. Successful application of these strategies in health has required an understanding of what value assessment entails, including quantifying the impact of a provider’s offering on patients’ and payers’ costs, as well as health gains. However, the adoption of value assessment in health has been slow, gradual, and multifaceted. One major explanation for this is that it required a paradigm shift “from patient to health consumer” to accept that some marketing concepts regarding customers are applicable to patients.

The idea of a paradigm shift toward a consumer-focused health system is not new. It was described as “taking hold” as early as the 1990s [1], with citations dating well into the 1980s—more than 4 decades ago. Much of the argument for a consumer-focused health care system revolved around changes in patients’ desire for treatments that provided value on their terms, rather than the health care systems’, with a desire for treatments that focused on functional loss, anxiety, and pain rather than traditional biomedical outcomes. The path to a consumer-focused health care system was not as easy or straightforward as envisioned 35 years ago, but the movement now has a push from changes in health care, ranging from an emphasis on patient satisfaction in pay-for-performance programs [2] to a push for payment transparency to empower consumer choice [3] to the push for open access to electronic health records for patients [4]. In the United States, there has also been a rise in high-deductible health plans [5], providing extra motivation for consumers to consider their health care purchases carefully [6].

Today, this paradigm shift seems to finally be taking hold, with the aid of better data and increased use of digital tools since the COVID-19 pandemic [7], which can be observed in the way health technology assessment (HTA) methods are elaborated, with more room for individual patient preferences. This paper reviews 20-25 years of value assessment approaches in health and the role of the internet, mobile technologies, and digital health. It first describes the foundation of value assessment in health. In the second part, it discusses HTA approaches and the development of the literature, and in the third part, it describes policy efforts and future opportunities for individual value assessment.

## Foundations of Value Assessment in Health

Value assessment uses tools to support better value in health care. The key question is value for whom. Many of the current value assessment tools used in health lack a sufficient patient-centered approach. Historically, most value assessment is based on the assumption that patients or “health consumers” maximize utility. In fact, the utility maximization paradigm forms the basis of many economic, psychological, cognitive, and behavioral models. Value assessment is performed by estimating a utility function that represents consumers’ preferences that are assumed to be complete and transitive. This means consumers can compare any 2 goods or services, and their preferences are internally consistent. Consumers can then rank-order the goods or services under consideration according to their personal “value function.”

When making a decision under certainty, the value function reflects the decision maker’s preferences on a particular outcome. Outcomes are defined by the assignment of values to a set of attribute variables that are either discrete or continuous and together make up for the space of all possible outcomes. However, outcomes are often not defined under certainty but in terms of probabilities, and a utility function is needed to assess the value of a decision. Expected utility theory (EUT), proposed by von Neumann and Morgenstern in 1944 [8], described a more complex utility function representing the decision maker’s attitudes regarding risk and the value of the outcomes by inducing a preference ordering on the probability distributions over the entire outcome space. Decades later, Kahneman and Tversky [9] contrasted the EUT concepts of risky and riskless choices with prospect theory (PT) [9]. Although the EUT assumed that risk attitudes derive exclusively from the way in which people value rewards as reflected in the curvature of the utility function, PT posited that one’s risk attitude would also vary with their subjectively weighted outcome probabilities.

To collect preferences and derive the utility function, there are many ways to ask consumers to rank-order options. In marketing research, some of the more established methods include contingent valuation [10], contingent ranking [11], contingent behavior [12], paired comparisons [13,14], discrete choice experiments [15], (full profile) conjoint analysis [16], adaptive conjoint analysis (ACA) [17,18], and ACA’s self-explicated prior [19]. In health, many of these methods have been adopted to define value for individual patients, often following the same patterns of discussion and methodological improvements as other fields. In all these methods, consumers are asked to implicitly or explicitly rank-order options and make choices based on the ordering. For example, in the ACA self-explicated model, consumers are asked to rank-order levels of an attribute of a choice, which is either assumed to be homogeneous across respondents or derived individually [19]. Next, they rate the importance of the difference between the best and the worst levels of an attribute on a 4-point scale, which is then multiplied by the preference orders and rescaled so that the difference between the highest and lowest partworths of an attribute is equal to the attribute’s importance [20].

In the self-explicated model, the choice set is truncated to 4 importance scores across, and the assumption of equal successive intervals within attributes is embedded in the model. This is in contrast to actual choices, where the choice set may have many attributes, such as price, color, quality, reliability, reputation, size, calories, and packaging, and also where some choices could be quite similar and others quite different, such as the choice between 2 kinds of sliced bread, a bagel, and a pita. In the case of paired comparisons, such as full-profile ACA, it is assumed that consumers sum the weighted additive differences between alternatives on each attribute [21]. In most preference elicitation methods, the values assigned to the attributes are discrete.

There are a number of practical challenges with these approaches. Issues with the reference point [22,23], scale [24], differences in attribute processing and heuristics [25,26], learning and fatigue effects [27], attention [28], and other challenges have caused considerable methodological discussion and new modeling approaches in health, marketing, transportation, and environmental economics over the past decades as researchers work to model consumer decision-making.

## Health Technology Assessment

Among the first publications in the past 25 years were studies focused on how consumers search for and appraise information on medicines on the internet. A qualitative study in 2003 found that many patients had a limited awareness of how they found and evaluated internet-based information on medicines [29]. An observational study using a convenience sample of 14 students published the same year focused on the proportion of adolescents finding correct or useful answers on the internet [30] and found that in about two-thirds of internet searches, they were successful in finding relevant health information.

In the early 2000s, most studies regarding decision-making focused on how patients gather information to make informed choices and how they could potentially use that information. This led to the formal concept of informed decision-making, which mostly entailed that patients would decide to undergo testing or treatment with them knowing and understanding the value or need for it, such as in the case of a study evaluating the effect of the web-based prostate-specific antigen (PSA) decision aid, Prosdex, on informed decision-making [31]. This study, published in 2010, was a web-based randomized controlled trial among men aged 50-75 years who had not previously undergone a PSA test, who were randomly allocated to 2 intervention and 2 control groups [31]. Participants in the intervention groups either viewed the decision aid Prosdex or were given a paper version of the text. The study showed that Prosdex is associated with improved knowledge about the PSA test and prostate cancer and that men who have a high level of knowledge have a less favorable attitude toward and are less likely to undergo PSA testing. Around this time, more studies used quantitative methods to assess the effect of health information on decision-making regarding health services and health behaviors. Examples include a study on the effects of internet-based tailored advice on the use of cholesterol-lowering interventions [32] and a feasibility study of an internet-based education and decision program for patients with early-stage prostate cancer [33].

In the years following, increasingly more studies appeared that discussed informed decision-making in the context of cost-effectiveness and cost utility [34-38], where the assessment of utility or value was now described as HTA. HTA is used to ensure that health care decisions consider relevant evidence about the costs and benefits of a treatment in a systematic way [39].

The studies mentioned focused primarily on the effect of digital technologies that have made an impact on decision-making. In general, new health technologies largely affected clinical and economic outcomes, and research methodologies to evaluate the efficiency of these new technologies were necessary. Cost-effectiveness analysis (CEA) is one way of doing this, which assesses the value or benefit of new health care technologies or interventions and compares the costs to a reference or threshold. In the United States, in the 1990s, the first and second panels on CEA included economists, ethicists, psychometricians, and clinicians who were asked to make reference case recommendations. The 1996 consensus report *Cost Effectiveness in Health and Medicine* was the first to describe the uses and conduct of CEA as a decision-making aid in the health and medical fields [40]. HTA was initially more actively developed in Europe in the 1970s, with both formal and informal initiatives in different countries [41]. So-called consensus conferences played an important role in the early development of HTA and had the explicit goal of using the best-available evidence in making policy, administrative, and clinical decisions in health care. By generating a broader interest in scientific evidence among policymakers, clinicians, and the general public, the conferences created a wider understanding of the need for comprehensive assessment in health care [42]. Although US policies have from there onward prevented the use of CEA for reimbursement decisions, such as with the Affordable Care Act of 2010, which banned Medicare from using CEA metrics, formal agencies were established in many individual European countries for HTA, starting with the first in Sweden in 1987, followed by many others in the 1990s.

n Australia, too, HTA of nonpharmaceutical technologies was developed in the 1970s and in 1990 for pharmaceuticals. It was established by the Commonwealth Parliament in the light of increasing costs of medical investigations and patient care [43]. Since almost 70% of total health expenditures in Australia are funded by government programs, including Medicare and the Pharmaceutical Benefits Scheme, evaluation was undertaken to consider the effects of developments in technology on medical benefits and public hospital costs, with some emphasis on diagnostic methods [43]. Economic evaluation is undertaken by or on behalf of the manufacturing industry, and the evidence on clinical effectiveness, safety, and cost-effectiveness presented is then considered by the Pharmaceutical Benefits Advisory Committee (PBAC).

### Quality-Adjusted Life-Years

HTA commonly uses evaluation methods to assess whether the costs per quality-adjusted life-year (QALY) gained from a treatment, pharmaceuticals, or intervention are within the conventional range of acceptability. QALY is a standard measure of the disease burden that includes both the quality and the quantity of a human life and is used in economic and health policy evaluation to assess the value of medical interventions [44]. One QALY equals one year of perfect health. This differs from a year of life gained, which does not consider the quality of life (QoL).

QALYs have been in use for a half century, with criticisms of the approach nearly as old [45]. Among the many criticisms of QALYs are challenges with incorporating equity [46], challenges in relating QALYs to economic models of utility [47], and questions about the valuation and the basis for weighting of different outcomes [48]. Despite these criticisms, QALYs are the standard in many countries for HTA.

Most HTA-oriented countries base their reimbursement model on a threshold number per QALY saved. Cost-effectiveness thresholds (CETs) vary widely by country and are typically used to assess whether an intervention is worthwhile from a country-specific standpoint and often reflect the perceived health opportunity cost [49]. For example, in Australia, the PBAC is believed to apply a threshold range of AU $45,000-$60,000 per QALY [50], it is GBP 20,000-40,000 per QALY in the United Kingdom, and in many European countries, this is between euros 50,000 and 100,000 per QALY saved. CETs used by other decision makers (eg, the World Health Organization’s suggested CET of 1-3 times the gross domestic product [GDP] per capita) do not. This way of calculating cost-effectiveness has been applied to various treatment options and conditions. Even internet-based interventions, such as those for harmful alcohol use, have been evaluated using this framework. A study evaluating the cost-effectiveness and cost utility of internet-based interventions for harmful use of alcohol [51], through the assessment of the incremental cost-effectiveness of IT compared with information systems (IS), found that the median incremental cost-effectiveness ratio was estimated at euros 3683 per additional treatment responder and euros 14,710 per QALY gained. At a willingness to pay euros 20,000 for 1 additional QALY, IT had a 60% likelihood of being more cost effective than IS.

The quality adjustments in QALYs are based on various measurement methods and instruments. One key early debate was whether “health” could be measured as a single index value or whether health should be represented by a series of values representing different aspects of health, such as physical, mental, and emotional health. In the late 1980s, an interdisciplinary 5-country group developed the EuroQol instrument, a 5D, 3-level generic measure subsequently termed the “EQ-5D” [52]. It was designed to measure and value health status in terms of 5 dimensions: mobility, self-care, usual activities, pain/discomfort, and anxiety/depression [53]. The valuation method expanded usage across clinical programs, disease and condition areas, population surveys, patient-reported outcomes, and value sets.

The EQ-5D has become the standard measure for health and provides a generic measure with a single index value representing health. The EQ-5D was designed to measure decrements in health across conditions and populations. Digital versions have been developed over the years, validated [54], and applied to various questions [55-58] regarding cost-effectiveness of interventions and treatments.

US policymakers have consistently chosen not to use the costs per QALY framework for reimbursement decisions. Indeed, in both the Affordable Care Act and the recent legislation authorizing Medicare to negotiate drug prices, the use of QALYs for value assessment was explicitly prohibited [59]. In recent years, the United States did see steady growth in the number of organizations conducting value assessments. National organizations, such as the Institute for Clinical and Economic Review (ICER) [60], the Innovation and Value Initiative (IVI), and the National Comprehensive Cancer Network (NCCN), have introduced value assessment frameworks and tools to guide health care decision makers in evaluating the relative benefits and costs of health care interventions, primarily pharmaceuticals. According to a recent analysis, the field of value assessment in the United States is dynamic and evolving [61], and the use of value assessment is gaining traction with health care decision makers and policymakers. Overall, 79% of surveyed health care payers reported that ICER recommendations influenced their decision-making in 2022, compared to only 49% in 2016 [62,63].

As health care decision makers and policymakers in the United States increasingly use value assessment tools to inform their decision-making, there are a variety of different tools that can be used, with different approaches to measuring value, endpoints, and criteria for assessing the different approaches [59]. DiStefano et al [59] suggested that these criteria could include feasibility, flexibility, and the ability to incorporate factors beyond the traditional value elements. They also contrasted measures that use conventional (QALY) endpoints with those that incorporate patient-centric value elements.

Patient-centered methods will not necessarily always align well with the averages that HTA in Europe often uses to make decisions on the use of a technology at a group level. Differentiation in the use of technologies for different groups of patients may not only be desired but also be more affordable if it means that some groups of patients prefer cheaper or no treatment. Patient-centered care means patients should be part of the decision-making process, not only through providing information, but also through providing authority to make decisions about their treatment plans and care path. Indeed, this is one of the reasons patient advocates in the United States have (to date) successfully lobbied against the use of QALY measures [45].

One example of this could be the treatment of prostate cancer. Treating prostate cancer carries the risk of impedance and may not meaningfully impact life-years; however, not treating the cancer carries the risk that the cancer metastasizes. The “correct” treatment will need to weigh the patient’s preferences for not being impendent versus the patient’s willingness to accept a cancerous tumor growing in their body versus expected life expectancy. In this example, the “average” best course of treatment may not provide guidance for the best course of treatment *for a particular patient*. Today, many patients with prostate cancer are consulted about their options and the advantages and disadvantages of each treatment path and are allowed to select their preferred treatment path.

Another example would be single versus double mastectomy. The decision on whether to remove a healthy breast in a woman with a high risk of breast cancer is not simply about QALYs gained or lost; it has profound personal implications around body image anxiety and fear of recurrence [64].

### Alternative CEA Approaches

There have been sporadic attempts to incorporate HTA into public health coverage decisions in the United States. The most well known is the attempt by the state of Oregon to use HTA principles to determine Medicaid coverage decisions. The state rejected standardized CEA and instead used a combination of expert opinion and public panels to create rank-ordered priorities. Oregon’s attempt to use CEA failed because of a combination of factors. One was a lack of public acceptance of the priorities; one of the most visible elements was a child, Adam Howard, who died after he was denied a bone marrow transplant because of the combination of a low probability of success and high cost. The second factor was the Americans with Disability Act (ADA), which both Bush and Clinton administrations argued was violated by the use of HTA.

Although CEA has not been well accepted in the United States, there are newer approaches that seek to incorporate HTA into decision-making in the United States. CEA has been supplemented with approaches to assess value, including multicriteria decision analysis (MCDA), generalized risk-adjusted cost-effectiveness (GRACE) analysis, and the “value flower.” MCDA is a decision-making method that systematically weighs various value elements that may fall outside traditional value assessments, such as a treatment’s scientific novelty, a patient’s disease severity, or how a treatment may affect a caregiver’s productivity [65]. MCDA models allow users to choose their willingness-to-pay thresholds and customize their value determination results using modifiable inputs for measures related to a treatment’s benefits and cost. There are an increasing number of studies reporting that defining the value of health care depends heavily on the decision context and stakeholders involved [66]. Where cost-utility analysis and QALYs have become the method and value definition of choice for traditional value judgements in coverage and pricing decisions, other criteria that may influence value are often not measured and therefore omitted from value assessments or are only used to qualitatively contextualize assessments. Recent qualitative research shows that for payers, value equates either with criteria that provide tangible benefits (from their perspective), such as new treatment options that respond to a serious unmet need. For patients, a population-level value equates to options that would potentially benefit them in the future and the value of hope. One suggestion made by Campbell et al [67] is to include the equal value life-year (evLY) as a measure of health gain that can be used as an alternative or a complement to QALY to address concerns related to undervaluing treatments that extend the life of individuals with serious illness or chronic disability. Measurement methods no longer solely insist on linear, additive utility. A systematic literature review [68] identified 15 relevant value frameworks and MCDA tools. These studies included a large number (n=56) of individual value criteria. The most commonly included novel criteria were an unmet medical need, severity of disease, and reduction in uncertainty. The identified scoring functions (measurement methods) for novel criteria were highly heterogeneous and tailored. Standardized scoring functions were not observed.

One example is GRACE analysis, which is a somewhat different approach to CEA that aligns economic assessments of treatments with patient preferences and experience of care [69]. In GRACE analysis, differential CETs (relative to traditional CEA) are applied based on disease severity (eg, higher thresholds for more severe diseases) and other patient circumstances to better recognize the value of treatments that promote equity and significantly improve patient QoL [69]. This links value assessment more tightly to economic theory, recognizing that the utility value of a health improvement varies depending on the underlying level of health. This was one of the key problems with the use of HTA in Oregon: treatments with acceptable QALY values but low utility values, such as routine dental care, were prioritized over conditions with lower QALY values but (potentially) higher utility values, such as bone marrow transplants. Although the GRACE method has been described at length in the literature [70-73], studies are now underway applying it and comparing it with different approaches and outcomes.

In addition to addressing potential nonlinearity of utility functions, there have been several initiatives to expand the set of “value measures.” A special task force of ISPOR, the professional society for health economics and outcomes research worldwide, created a “value flower” that identified elements of value that are and are not typically included in standard CEA and a recommendation to expand cost-effectiveness measures with these value elements to better capture what is important to individuals but unmeasured in standard QALYs [74]. In general, newer approaches to CEA now seek to include measures of productivity, real option value, insurance value, reduction in uncertainty, scientific spillover, severity of disease, adherence-improving factors, equity, and the value of hope. The issue with all these novel approaches, however, remains that the ultimate goal is to draw conclusions about *societal* costs and benefits rather than a blueprint for *individualized* health care delivery based on patient preferences and perceptions.

## Policy Efforts and Future Opportunities

### Patient Centeredness

In the policy arena, a series of important changes have been made regarding the patient’s position in the decision-making process. For decades, there have been attempts to improve “patient centeredness” in health care, which is defined as “providing care that is respectful of and responsive to individual patient preferences, needs, and values and ensuring that patient values guide all clinical decisions” [75]. Although the focus on patient-centered care has increased, the rationale, measurement, and implementation of strategies to improve patient-centered care or to use patient experiences for quality improvement purposes have been widely debated [76,77]. In practice, patients are not directly and proactively involved as much in the process of quality improvement. Even if efforts to design care through the patient’s eyes [78] are supported, the question remains to what extent this can be done at the individual patient level. Information and communication technologies have more recently shown to be able to support chronic disease self-management, but this self-management should be accompanied by a shift in focus on integrating the patient’s own perceptions of the chronic disease and the health care system’s approach to managing it [79]. As mentioned previously, we therefore need to use and improve methods to assess patients’ perceptions, attitudes, beliefs, and preferences for health services and self-management.

Various patient-reported outcomes have since been developed and evaluated, with most studies concluding that health care professionals and patients alike need to choose the most suitable patient-reported outcomes for their patients, which may not be the same for every patient population or individual [80]. For health insurance, for example, the Health Plan Employer Data and Information Set (HEDIS) has been used to compare and report quality across health plans from a patient perspective, but in a number of cases, these do not appear to be cost effective or provide value for certain subgroups [81].

### Shared Decision-Making

The same is true for the concept of shared decision-making, a process by which patients and providers consider outcome probabilities and patient preferences and reach a health care decision based on mutual agreement [82]. This is best used in situations in which there is medical uncertainty or different treatment options. Theoretically, both provider and patient discuss the different options and reach a conclusion together about the optimal strategy. In the United States, for example, policy initiatives to improve shared decision-making were embedded in both the Affordable care Act (2010) and the patient-centered medical home, but their success depends on building a good relationship so that information is shared and patients are supported to deliberate and express their preferences and views during the decision-making process. Increasingly more (codesigned) decision aids have been developed in recent years to support shared decision-making and provide room for individual preference heterogeneity. These decisions aids are also being evaluated, with many studies concluding that when designing interventions to improve health outcomes, it is important to consider this heterogeneity. For example, a study looking at a decision aid for diabetes self-care found that teenagers with type I diabetes mellitus prioritize reducing family conflict and fitting into their social milieu over health outcomes at this time in their lives [83]. Another study focused on gaps in the current approach to shared decision-making for children with medical complexity suggested that clinicians should focus decision-making discussions on integrating each child’s unique situation, the insights parents gain through their decision-making activity, and their clinical knowledge to enhance the understanding between parents and health care providers beyond the narrow concept of parental values [84].

In the United Kingdom, health authorities have engaged clinical champions and patient representatives in national initiatives for shared decision-making and embarked on a process of widely disseminating patient decision aids [85]. In practice, however, providers often find that the time required to go over all necessary information and decision aids in not available. Decision aids ultimately suffer from the same challenge as the concept of patient centeredness; there is not enough room in the clinical care pathway for approaches that incorporate individual preferences.

## Future Opportunities for Value Assessment

Patient value is a key component in all areas of health care delivery, and understanding how health providers create, communicate, and deliver value to patients is a key factor when seeking ways to improve care according to the triple aim of improving the individual experience of care; improving the health of populations, and reducing the per capita costs of care for populations [86].

The practice of HTA uses a societal perspective to measure the cost-effectiveness of treatment in terms of costs and QALYs, where QALYs assume that a health state that is more desirable is more valuable, and therefore, value is equated with preference or desirability. This approach has major empirical and conceptual shortcomings, such as inconsistencies among values obtained from the standard gamble, time trade-off, and visual-analog scale elicitation formats and, more importantly, the linearity assumptions that violate the key economic assumption of diminishing marginal utility. Although HTA has come a long way in its “multifaceted assessment of the clinical, economic, ethical, legal, and societal perspectives that may be impacted by a new technology, procedure, drug, or process” [87], these approaches may not sufficiently reflect individual patient or health consumer preferences that may be important in the assessment of “value” in the current value-based health care discussion.

The key difference in measuring value is that the outcome in other fields is an economic and tangible measure, such as purchasing a car, whereas in health, it is the production of health. One way to better predict behavior and choice in health, therefore, is to include the health production function. Our recent work on medication nonadherence, for example, has shown that there is an enormous discrepancy between patient preferences and their beliefs and expectations [88]. In a pilot study using a double-bound contingent-belief (DBCB) questionnaire, patients could express how efficacy and side effects are affected by controlled levels of nonadherence, allowing for the estimation of sensitivity in health outcomes and costs. The derived health production function suggested that patients may strategically manage adherence to minimize side effects without compromising efficacy. Patients’ inclination to manage medication intake was closely linked to the relative importance they assign to treatment efficacy and side effects. We formalized the definition of health production functions by positing that patients craft a mental model of health production under scenarios that are not covered by interactions with their physicians or by the clinical evidence available to them. This function relates the benefits of health behaviors to the costs associated with achieving such benefits. Value, then, is now defined not only by preferences for health outcomes alone but also by the patient’s expectations of health outcomes. The “mental model” described in this paper is now being tested in different studies.

In addition to tangibility, a second key difference is that the production and consumption of a medical service take place simultaneously. This inseparability and the inconsistency in health that is being produced give much more room to interpretation, experiences, perceptions, and emotions when making a decision. In some cases, patients may be informed regarding clinical efficacy, but they may care more about another attribute of service or treatment that is entirely unrelated to clinical outcomes but not irrelevant to them. For example, a physician advises a patient to wait with treatment because the patient is at a risk of side effects at that point in time. The physician is attempting to provide high-quality care and act in the patient’s best interest, while maintaining a reputation for delivering high-value care. This particular patient may choose immediate treatment because the gains outweigh the potential side effects and because the patient does not care about the physician’s reputation. This creates a conflict between QALY-type HTA assessment and individual assessments of “value.”

In a situation like this, who defines what “value-based” care means? If policymakers truly want to promote patient-centered care and shared decision-making, the question is what the desired outcome is in this situation. Reimbursement decisions are currently based on an equation that defines value in terms of health states. We know that this approach tells only one part of a story—what is preferable from a societal approach. If we accept the notion that all patients are different and have different preferences, it must be acknowledged that “value” in health care needs to be redefined to incorporate patient preference heterogeneity.

Value assessment in health has come a long way in the past 25 years. Currently, health economic models focus on the benefits and costs of treatment options and help define reimbursement and delivery systems. However, this top-down approach needs to make room for “value-based” care, which considers value to individual patients. Value-based care ties the amount health care providers earn for their services to the value they deliver for their patients, such as the quality, equity, and cost of care. Through financial incentives and other methods, value-based care programs aim to hold providers more accountable for improving patient outcomes. However, in practice, so far, the focus is primarily on cost containment.

In this system, there is little attention given to variations in individual patient preferences or factors that affect those preferences. Many value-based health care systems still focus on cost containment strategies for the average patient, not the individual value to individual patients [89]. The idea is that some treatments, such as some imaging for low back pain, should not be provided (to anyone), while other treatments, such as vaccines, should be uniformly dispensed (to everyone). The one-size-fits-all approach to value disproportionally makes some patients worse off. In addition, delivery systems have differential effects on patient populations, which further enhances health inequities. Theoretically, it also increases spending, because all patients are expected to require a treatment for a weighted average cost. In practice, some will require higher-cost treatment options, while others will value lower-cost options. Care delivery that considers all aspects of human decision-making will help pave the way for more accurate cost-benefit analysis and improved access and affordability.

One lesson learned from 25 years of value assessment in health is that there is no average patient, yet many HTAs are still based on averages. That is why value assessment from a societal perspective may lead to equitable allocation of resources without necessarily leading to optimal use of resources. If individual patient behaviors and preferences are not considered in value assessment methods, resources will be wasted because of a mismatch between what the consumer values and what society prioritizes.

It will be challenging to consider the complexities of individual preferences and behaviors, especially if they are not met at the societal level. There has been an increase in the adoption of ideas from behavioral economics and mathematical psychology to better understand human decision-making in health. There are ways to add information about cognitive and brain processes that may help value assessment in health to perform more precise predictions of human decision-making and choice.

However, the final challenge for future value assessment will be to incorporate heterogeneity of preferences into decision models. Currently, heterogeneity is largely considered in terms of health equity and the impact of approval of denial of treatment on disadvantaged populations. However, the more challenging aspect will be trying to reconcile heterogeneous patient preferences with decision tools based on “average” patients. Centralized decision makers using average metrics, such as “incremental cost-effectiveness ratios,” are reliant on the strong assumption of linearity of preferences, which is unlikely to hold in heterogeneous/diverse societies. This means that centralized planners using decision rules based on measures such as the average cost per QALY gained will misallocate resources and fail to use health care dollars to maximize the well-being of society.

## Conclusion

Where ultimately the current energy toward developing new measures of value will lead is unclear. Clearly, patients are interested in better measures of value, but whether any of the new measures that have been developed will be meaningful and useful to consumers is unknown and a fruitful area for future research. Similarly, patient advocates will tend toward broader measures of value, but whether the rank-ordering of treatments will ultimately change is unknown—and another fruitful area for future research. Finally, how central payers will incorporate the new measures into their decision-making is perhaps the biggest unknown of all. Research will need to demonstrate the value of the new measures in providing better measures of value that meaningfully change health spending priorities.

## Abbreviations

ACA: adaptive conjoint analysis
CEA: cost-effectiveness analysis
CET: cost-effectiveness threshold
EUT: expected utility theory
GRACE: generalized risk-adjusted cost-effectiveness
HTA: health technology assessment
ICER: Institute for Clinical and Economic Review
IS: information systems
MCDA: multicriteria decision analysis
PBAC: Pharmaceutical Benefits Advisory Committee
PSA: prostate-specific antigen
PT: prospect theory
QALY: quality-adjusted life-year
QoL: quality of life
